# Primary Pleuropulmonary Synovial Sarcoma: A Case

**DOI:** 10.1155/2018/5190271

**Published:** 2018-04-04

**Authors:** Fatima Zahra Mrabet, Hafsa El Ouazzani, Leila El Akkari, Sanaa Hammi, Jamal Eddine Bourkadi, Fouad Zouaidia

**Affiliations:** ^1^Department of Pneumology, Moulay Youssef University Hospital Center, Rabat, Morocco; ^2^Faculty of Medicine and Pharmacy, Mohammed V University, Rabat, Morocco; ^3^Department of Pathology, Ibn Sina University Hospital Center, Rabat, Morocco

## Abstract

Primary pleuropulmonary synovial sarcoma is extremely rare. The diagnosis can only be made after having eliminated an extrapleuropulmonary localization in the past and at the time of diagnosis. Our presentation is about a 40-year-old woman having a cough and dyspnea since three weeks ago; imaging had showed a left pleurisy with pleuropulmonary process. Histological study of the biopsy confirmed the diagnosis of pleuropulmonary synovial sarcoma. PET-SCAN had not identified any extrathoracic localization. This tumor is known for its aggressive nature and high risk of metastasis. Its primitive character is retained following a diagnostic procedure of exclusion. Surgical treatment remains the best therapeutic tool when it is technically feasible; otherwise the prognosis is often unfortunate. In this paper, we report a case of primary pleuropulmonary synovial sarcoma. Through this case, we present a rare disease that is often difficult to diagnose.

## 1. Introduction

Synovial sarcoma is a rare soft tissue sarcoma accounting for 8% of all soft tissue tumors in the body. It is not originating from the synovial tissue, but is arising from pluripotent mesenchymal tissue. In lung, metastatic synovial sarcoma from extremities is the most common in pulmonary parenchyma and pleura [1].

Primary pleuropulmonary synovial sarcoma is a very rare, but highly aggressive, malignant neoplasm. It must be differentiated from other spindle cell tumors that have similar morphological features through the immunohistochemical study [2].

## 2. Case Report

**Figure 1:**
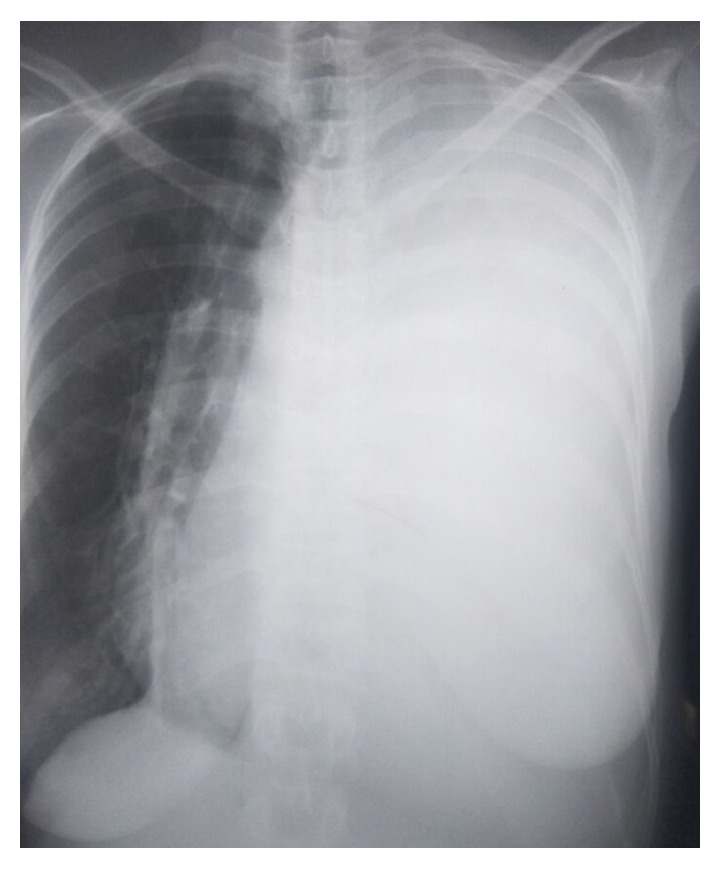
Posteroanterior chest roentgenogram showing a homogeneous opacity taking the whole hemi thoracic left field and pushing the mediastinum towards the contralateral side.

Mrs. M. K. a 40-year-old woman, with no notable pathological history, just gave birth a month ago; she has had left chest pain, dry cough, and stage III of mMRC dyspnea evolving, since three weeks ago before her admission, in a context of deterioration of the general state. Clinical examination revealed a left fluid effusion syndrome. The posteroanterior chest roentgenogram showed a homogeneous opacity occupying the totality of left thoracic field with the presence of signs of mediastinal discharge in the right side (Figure 1). An evacuation of the pleurisy was performed repeatedly to relieve the patient (about 2 liters every 3 days).

**Figure 2:**
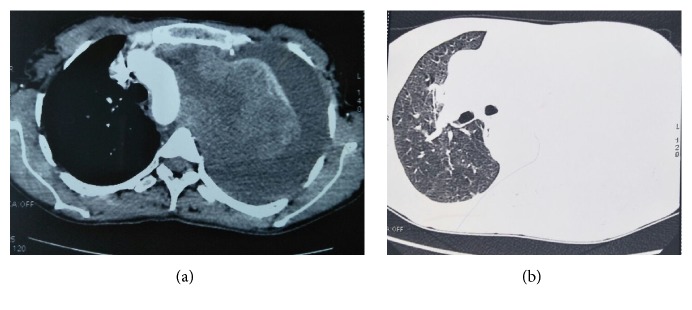
CT scan image showing a left mediastinal-pulmonary tumor process [(a) mediastinal window. (b) Parenchymal window].

Thoracic ultrasonography confirmed the presence of pleurisy of high abundance and guided the pleural biopsy returned inconclusive. The thoracic CT showed a mediastino pulmonary process localized at the left lobe superior measuring 104 *∗* 102 *∗* 141 mm comes in contact with the artery under Clavier and the left lateral edge of the aorta and surrounded the left branch of the pulmonary artery which stayed permeably associated with pleurisy ipsilateral of high abundance (Figure 2). Bronchial fibroscopy showed a bud in the left upper lobe bronchus with thickening of its spurs, but the histological study was not contributive twice.

**Figure 3:**
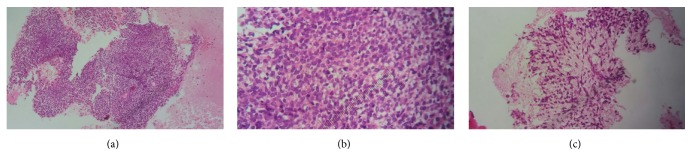
Histologically, (a) the tumor showed spindle-shaped cells forming sheets (Hematoxylin Eosin GX10), (b) the spindle cells are of uniform appearance with oval, dark-staining nuclei and scanty amount of indistinct cytoplasm (Hematoxylin Eosin GX20), and (c) we noted a myxoid pattern (Hematoxylin Eosin GX10).

**Figure 4:**
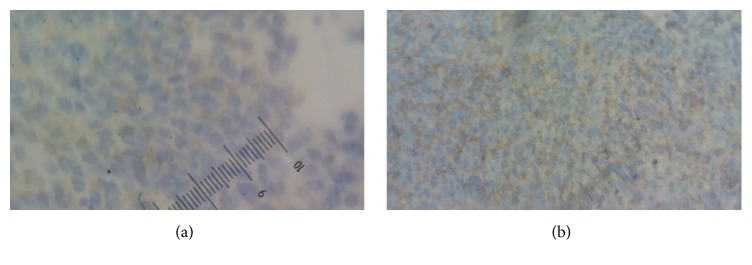
On immunohistochemical study, tumor cells were positive for EMA (a) and CD 99 (b).

Transthoracic biopsy of the pulmonary tumor process was performed (guided by CT) by using the biopsy needle Gelman type (18 G 11 cm). It is concluded from grade II synovial sarcoma of the FNCLCC at the histological study of the fragments. The tumor showed spindle-shaped cells forming sheets. The spindle cells are of uniform appearance with oval, dark-staining nuclei and scanty amount of indistinct cytoplasm (Figure 3). On immunohistochemical study, tumor cells were positive for EMA and CD 99. They were negative Cytokeratin AE1/AE3, Desmin, PS 100, and CD34 (Figure 4).

The extension assessment did not show extrathoracic localization, especially a PET SCAN, which did not show extrathoracic hypermetabolism, particularly in the soft tissues, which is, a priori, a fundamental element of the primitive character of the tumor. Unfortunately we could not perform chromosomal studies on this patient; we lack the molecular platform in our instruction because we belong to a low-income country. We retained the diagnosis after eliminating the differential diagnosis.

After the confirmation of the diagnosis, the patient was referred to the oncology center for chemotherapy and died the day after her first cure of treatment.

## 3. Discussion

Synovial sarcoma is a rare tumor of the young adult. It is located in 90% of the cases in the para-articular regions and in 10% of the cases in various anatomical sites not related to the synovial tissue [3].

Primary pulmonary sarcomas are very rare and comprise only 0.5% of all primary lung malignancies with only a few case reports in the literature. Primary pleuropulmonary and mediastinal synovial sarcomas are more aggressive than soft tissue synovial sarcomas with rare distant metastasis. The diagnosis of primary pleuropulmonary synovial sarcoma (PPSS) requires a combination of clinical, radiological, pathological, and immunohistochemical investigations to exclude alternative primary tumours and metastatic sarcoma [4].

The average age of onset for pleuropulmonary location is 38.5 years. It is not related to cigarette smoking. It affects both sexes with similar incidence on the right and left lungs [4, 5].

Our patient is a nonsmoking woman, and she is 40 years old.

The origin tissue of synovial sarcomas is not well defined; it appears that they develop from a pluripotent mesenchymal cell with synovial differentiation [6]. The term “pleuropulmonary” was first recommended by Essary and colleagues to describe the anatomic subset of primary synovial sarcomas originating from either the lung or the pleura due to inherent difficulties in assigning a precise anatomic origin in most cases. There has been no large series documenting the exact number of repeated pleuropulmonary synovial sarcoma cases worldwide. Ipsilateral pleural effusion was reported, while mediastinal lymphadenopathy was rare [7]. Our patient presented pulmonary and pleural localization at the same time; the presence of an endobronchial bud at bronchial fibroscopy is more in favor of a pulmonary primitive; otherwise double simultaneous localization at the same density at thoracic CT makes it difficult to decide. As described in the literature, the chest CT scan did not show the presence of lymphadenopathy in our presentation, and the pleural location is ipsilateral with respect to pulmonary localization.

The symptomatology as well as the imaging is not characterized by specific sign directing towards a synovial sarcoma. The clinical and radiological data are that of a pleuropulmonary tumor process without histological specificity. So far, four cases of PPSS presenting during pregnancy have been reported [2]. Our patient also had a nonspecific clinical and paraclinical presentation occurring at her first month of postpartum.

Radiologically, compared with soft tissue synovial sarcoma, primary pulmonary and mediastinal synovial sarcoma show less vascularity and a similar “triple sign” (bright, dark, and gray) representing tumor, hemorrhage, and necrosis on magnetic resonance imaging. The presence of significant adenopathy, however, argues against PPSS [8].

The seat is preferentially peripheral but some cases are described at the level of the bronchial tree. The localization of the tumor process in our patient was mediastino-pulmonary with presence of a bud at the level of the lobar bronchus superior to fibroscopy.

Histologically, there are four histologic subtypes: biphasic, monophasic (spindle), monophasic epithelial, and poorly differentiated (round cell) tumors. The most commonly observed subtype is monophasic, and the biphasic subtype is easily diagnosed on the basis of the presence of both epithelial and spindle cells. However, the monophasic subtype can be misdiagnosed as other types of sarcoma [9]. Our patient had a monophasic fusiform subtype at the biopsy.

In the presence of malignant tumor proliferation of spindle-cell sarcomatous aspects, a carcinomatous component should always be sought to eliminate the diagnosis of sarcomatoid carcinoma. Once the pure character of sarcomatous proliferation is established, the possibility of a sarcomatoid carcinoma, which, unlike synovial sarcoma, is always rich in cytonuclear atypia, must be rejected at first sight. In addition, proliferating cells are intensely and diffusely positive to epithelial markers. After discarding these 2 more frequent events the diagnosis of sarcoma can be retained [10].

Other differential diagnoses include the fibrous pleural tumor, sarcomatoid subtype of malignant pleural mesothelioma, spindle cell carcinoma, and malignant peripheral nerve sheath tumor [10].

The next question is whether the pleuropulmonary tumor is primitive or secondary. The second eventuality is by far the most frequent. The mother tumor usually sits in the soft tissues. Only the absence of extrapleuropulmonary tumor localization in the past and at the time of diagnosis will attest to the primitive nature of the tumor. In our case, PET SCAN had eliminated an extrathoracic localization of the tumor, so we had retained the diagnosis of primary pleuropulmonary synovial sarcoma.

The recent identification of a chromosomal translocation specific to pleuropulmonary synovial sarcoma has increased the recognition of this particular sarcoma subtype. The chromosomal translocation t(x;18) (p11.2;q11.2) is present in more than 90% of cases of primary PPSS [11]. It produces three types of fusion genes formed in part by SS18 from chromosome 18 and by SSX1, SSX2, or, rarely, SSX4 from the X chromosome [12]. Unusual sites of involvement include the kidney, adrenal gland, retroperitoneum, lung, mediastinum, bone, and nervous system [11]. Despite its high sensitivity, molecular testing is not required if the diagnosis of synovial sarcoma is certain or probable on the basis of clinical, histological, and immunohistochemical evaluations [5].

There is no standardised therapy for PPSS and most patients are treated with surgery alone or surgery with adjuvant radiation therapy. Synovial sarcomas are chemosensitive to ifosfamide and doxorubicin, with an overall response rate of approximately 24 percent. A new drug, pazopanib, seems to provide another option with an improved median progression-free survival and median overall survival in some trials. Radiotherapy has no apparent effect on the control of local disease or overall survival [9, 13].

The prognosis for patients with PPSS is poor, with an overall 5-year survival rate of 50 percent. Factors predicting a worse prognosis for patients with synovial sarcomas include tumour size (>5 cm), male gender, older age (>20 years), extensive tumour necrosis, high grade, large number of mitotic figures (>10 per 10 hpf), neurovascular invasion, and, recently, the* SYT-SSX1 *variant [9]. In our observation, approximately 45 days between the appearance of the first clinical signs and the confirmation of the diagnosis, the patient died the day after her first chemotherapy treatment testifying to the aggressiveness of the tumor.

## 4. Conclusion

Primary pleuropulmonary sarcomas are rare. They first discuss a tumor with double carcinomatous and sarcomatous contingent whose first component is discrete or was not interested in sampling. Once the diagnosis of sarcoma is established, a secondary pulmonary site should be removed, which is more likely. It is therefore only after a diagnostic procedure of exclusion that the primary pulmonary seat of a sarcoma will be retained.

The feature of our observation compared to literature revues is the clinical, radiological, and histological similarity.

## Abbreviations

CT: Computed tomography
PPSS: Pleuropulmonary synovial sarcoma
FNCLCC: French Federation Nationale des Centres de Lutte Contre le Cancer
mMRC: Modified Medical Research Council
PET SCAN: Positron-Emission Tomography Scanning.
